# Prevalence and Antimicrobial Susceptibility Profile of *Staphylococcus aureus* in Milk and Traditionally Processed Dairy Products in Addis Ababa, Ethiopia

**DOI:** 10.1155/2021/5576873

**Published:** 2021-07-16

**Authors:** Fikirte Lemma, Haile Alemayehu, Andrew Stringer, Tadesse Eguale

**Affiliations:** ^1^Federal Ministry of Agriculture, Addis Ababa, Ethiopia; ^2^Aklilu Lemma Institute of Pathobiology, Addis Ababa University, Addis Ababa, Ethiopia; ^3^College of Veterinary Medicine, North Carolina State University, USA

## Abstract

*Staphylococcus aureus* is a contagious pathogen that can cause various diseases in both humans and animals. Antimicrobial-resistant *S. aureus* is becoming an extremely important global health problem. A cross-sectional study was conducted from December 2019 to May 2020 to assess the occurrence of *S. aureus* and its antimicrobial susceptibility profiles in milk and traditionally processed dairy products in selected subcities of Addis Ababa. A total of 255 dairy product samples (175 raw milk and 80 traditionally processed dairy products) were collected from farms and retail markets. Samples were cultured for *S. aureus* according to standard microbiology techniques, and the Kirby–Bauer disk diffusion method was used to assess antimicrobial susceptibility of isolates to a panel of 12 antimicrobials. Susceptibility to methicillin was determined based on the sensitivity of isolates to cefoxitin, and resistant isolates were investigated for the presence of *mec*A and *mec*C genes using PCR. *Staphylococcus aureus* was isolated from 43 (24.6%) of milk, 7 (17.5%) of yogurt, and 2 (5%) of cottage cheese. A significantly higher rate of contamination with *S. aureus* was recorded among milk samples compared to yogurt and cottage cheese (*p* = 0.019). Out of 52 *S. aureus* isolates investigated for susceptibility to 12 antimicrobials, 49 (94.2%) of the isolates were resistant to ampicillin and 42 (80.8%) to amoxicillin+clavulanic acid. Twenty (38.5%) of the isolates were methicillin-resistant *S. aureus* (MRSA) based on susceptibility to cefoxitin. However, only one of these isolates (5%) was positive for *mec*A gene, and none of them were positive for the *mec*C gene. There was no significant difference (*p* > 0.05) in the rate of occurrence of MRSA among isolates from different sources. In conclusion, this study demonstrated a significant level of contamination of milk and dairy products with *S. aureus* and most isolates were multidrug resistant. The occurrence of MRSA in raw milk and dairy products signifies a serious public health threat as the practice of consuming raw dairy products in the study area is widespread. The lack of agreement between phenotypic and genotypic detection of MRSA suggests the need for further study to identify the genetic basis for the observed resistance phenotype.

## 1. Introduction

Dairy products including milk have long been recognized as an important food for human physical and cognitive development due to the nutrients contained which are essential for growth and healthy development [1]. However, dairy products may contain pathogenic microorganisms and have a role in the transmission of these to humans [2]. *Staphylococcus aureus* is one of the most pathogenic bacteria isolated from milk. *Staphylococcus aureus* can be detected in milk due to contamination while milking or may originate from the milk obtained from cows affected by subclinical mastitis cases caused by *S. aureus* or due to post harvest contamination [3–6]. Subclinical mastitis caused by *S. aureus* and other pathogens was shown to seriously impact yield and composition of milk from dairy cows [7].

Milk contaminated with *S. aureus* can serve as a source of serious infections and staphylococcal associated toxins [2, 8]. Treatment of animals with clinical and subclinical mastitis with antimicrobials is commonly practiced to mitigate the economic and health consequences of mastitis in dairy cattle. However, the success of this therapy can be hampered by the high number of microorganisms resistant to certain antimicrobials due to their inappropriate use. In addition, antimicrobial-resistant *S. aureus* isolates and associated resistant genetic markers can be transferred to humans through the food chain, presenting additional public health concern [9]. Of all the resistance traits, methicillin-resistant *S. aureus* (MRSA) is clinically the most important, since MRSA isolates are resistant to most commonly prescribed class of betalactam antimicrobials [10].

Previous studies reported that 23.4% and 24.2% of raw cow milk samples were contaminated with *S. aureus* in central Ethiopia [11] and in north-western Ethiopia [3]. High rates of resistance to commonly used antimicrobials were also reported. For instance, Ayele et al. [11] reported that 100% of isolates were resistant to cefoxitin (methicillin), 98.5% to penicillin G, and 77.9% to streptomycin whereas Mekonnen et al. [3] reported 86% resistance to penicillin/ampicillin and 54% resistance to tetracycline; however, none of the isolates were resistant to methicillin. Understanding the prevailing situation of *S. aureus* in dairy products at various levels of production and their antimicrobial susceptibility is important to guide the appropriate use of antimicrobials in animals and humans as well as to devise possible alternatives to mitigate the burden of antimicrobial-resistant strains. Therefore, this study is aimed at investigating occurrence of *S. aureus* and antimicrobial susceptibility profile of isolates from milk and other dairy products in Addis Ababa, Ethiopia.

## 2. Materials and Methods

### 2.1. Study Area

**Figure 1:**
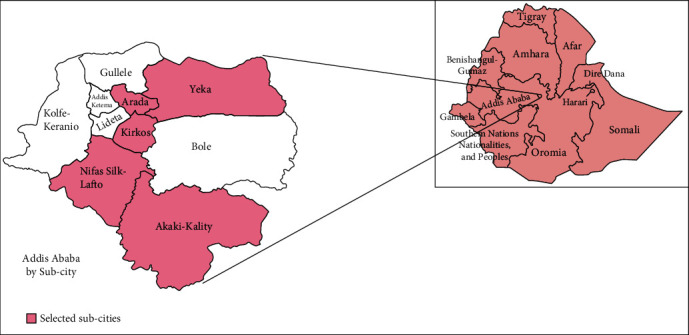
Map of Addis Ababa, showing the five subcitiess involved in the study.

The study was conducted in Addis Ababa, the capital city of Ethiopia from December 2019 to May 2020. Addis Ababa lies in the central highlands of Ethiopia at an altitude of 2350 m above sea level located at 9° 1′48^″^N and 38° 44′ 24^″^E. The average annual temperature in Addis Ababa is 16.3°C whereas average annual precipitation is 1143 mm [12]. The city is divided in to 10 subcities, of which the following five subcities were selected using simple random sampling technique for this study, namely, Akaki Kality, Nifas Silk Lafto, Kirkos, Yeka, and Arada (Figure 1).

### 2.2. Study Population

The study population was dairy farm owners in Addis Ababa who were currently producing milk and retail shops involved in selling traditionally processed dairy products (yogurt and Ethiopian cottage cheese) that are commonly consumed in the city. Urban dairy farms are contributing significantly towards filling in the large demand for dairy products in Addis Ababa.

### 2.3. Study Design and Sample Size Determination

A cross-sectional study was conducted from December 2019 to May 2020 to investigate the occurrence and antimicrobial susceptibility of *S. aureus* in milk and dairy products. Sample size was determined using the formula described previously [13] with 95% confidence, desired absolute precision of 5% and an expected 12.6% prevalence of *S. aureus* in raw milk from previous study [14] and 5% in dairy products (yogurt "Ergo" and cottage cheese "Ayib") [15]. This resulted in minimum sample size of 169 for raw cow milk and 73 for other dairy products. However, a total of 255 samples (175 raw milk and 80 other dairy products) were collected for greater study power.

### 2.4. Sampling Methodology and Sample Collection

The five subcities were selected using a simple random sampling technique. Representative “Woredas” (districts) (smallest administrative units) and dairy farms were then selected from these subcities using systematic random sampling technique based on the list of Woredas and dairy farm owners. The sampling frame used for selection of the dairy farms was a list of households registered by the Addis Ababa Farmers and Urban Agricultural Commission. The number of samples was proportionally allocated to each subcity based on the number of farms available in each Woreda. All raw cow milk samples were collected directly from dairy farmers, whereas traditionally produced yogurt and cottage cheese (*n* = 40 each) were collected from retail dairy product vendors in the study area. Inclusion of dairy product vendors was carried out by searching for shops selling traditionally processed yogurt and cottage cheese in specific Woredas during dairy farm visits. Every other shop along our way was included until the required sample size was fulfilled.

From each sampling unit, 25 ml of mixed raw milk from cows in a farm, 25 g of cheese, and 25 ml of each yogurt sample from dairy product vendors were collected in a sterile screw-capped bottles. Strict aseptic procedures were followed when collecting milk samples in order to prevent contamination. The bottles containing samples were labeled and transported to Microbiology Laboratory, Aklilu Lemma Institute of Pathobiology, Addis Ababa University, in ice box containing ice pack within 3-4 h of collection.

### 2.5. Bacterial Isolation and Identification

Upon arrival at the laboratory, 25 g/ml of each sample was diluted separately in a 225 ml of Tryptic Soy Broth (TSB) with 10% sodium chloride (Oxoid, Hampshire, UK) and thoroughly mixed. Samples were then incubated at 37°C for 24 h. A loopful of suspension was then streaked onto a Mannitol Salt Agar plate (Oxoid, Basingstoke, Hampshire, England) and incubated at 37°C for 24 h. Typical presumptive *S. aureus* colonies characterized with golden yellow pigmentation were then transferred into nutrient broth and incubated at 37°C for 24 h. The presumptive *S. aureus* isolates were confirmed using standard microbiological and biochemical tests: the Potassium Hydroxide (KOH) test, catalase, and coagulase tests. *Staphylococcus aureus* ATCC25923 was used as a reference strain.

### 2.6. Antimicrobial Susceptibility Testing and PCR-Based Detection of *mec* Genes

*Staphylococcus aureus* isolates were tested for their susceptibility to commonly used antimicrobials on Mueller Hinton Agar (MHA) (Oxoid, Hampshire, UK) using Kirby–Bauer disk diffusion method as described by the Clinical Laboratory Standards Institute guidelines [16]. Susceptibility of *S. aureus* isolates was tested for 12 antimicrobials, namely, tetracycline (30 *μ*g), amoxicillin-clavulanic acid (30 *μ*g), ampicillin (10 *μ*g), cephalothin (30 *μ*g), ciprofloxacin (5 *μ*g), ceftriaxone (30 *μ*g), gentamicin (10 *μ*g), cefoxitin (30 *μ*g), erythromycin (15 *μ*g), cefoxitin (30 *μ*g), chloramphenicol (30 *μ*g), and trimethoprim+sulfamethoxazole (1.25/23.73 *μ*g).

*Staphylococcus aureus* isolates were grown on Mueller Hinton broth for 4-5 h at 37°C, and its concentration was adjusted to 0.5 McFarland standards. The inoculum was evenly spread on MHA plate using sterile cotton swab, and antimicrobial discs were placed 15 minutes after bacterial inoculation. The plate was then incubated at 37°C for 24 h, and the diameter of zone of inhibition was measured to the nearest millimeter using caliper. The interpretation of the categories of susceptible, intermediate, or resistant was based on the CLSI guidelines [16]. Phenotypic identification of MRSA was based on resistance to cefoxitin [17]. Reference strain of *S. aureus* ATCC25923 was used as a quality control organism. Molecular detection of *mec*A and *mec*C genes was performed using PCR as previously described [18] using forward and reverse primer pairs: 5′-TCCAGATTACAACTTCACCAGG-3′ and 5′-CCACTTCATATCTTGTAACG-3′ and 5′-GAAAAAAAGGCTTAGAACGCCTC-3′ and 5′-GAAGATCTTTTCCGTTTTCAGC-3′, respectively. The expected amplicon size for *mec*A was 162 bp while that of *mec*C was 138 bp.

### 2.7. Statistical Analysis

Descriptive statistics were utilized to summarize the information collected in the different stages of this study. The prevalence of *S. aureus* in milk and dairy product samples was calculated, and the association between different risk factors with *S. aureus* sample positivity was assessed using the Chi-square test. Logistic regression was used to explore the assocation between type of sample and increased odds of being positive for *S. aureus*.

### 2.8. Ethical Consideration

Ethical approval to conduct this study was obtained from the Institutional Review Board of Aklilu Lemma Institute of Pathobiology (ALIPB), Addis Ababa University, with (Ref No.: ALIPB IRB/009/2012/20). Verbal consent was obtained from dairy farm owners and dairy product vendors after explaining the purpose and importance of the study prior to start of data collection. Participation of the dairy farmers and dairy product vendors was solely based on their willingness.

## 3. Results

### 3.1. Prevalence of *Staphylococcus aureus* in Raw Milk and Other Dairy Products

*Staphylococcus aureus* was detected in 43 (24.6%) of milk samples, 7 (17.5%) of yogurt "Ergo" samples, and 2 (5%) of cottage cheese “Ayib” samples. The prevalence of *S. aureus* was significantly higher in milk and yogurt samples when compared to cheese samples (*p* < 0.05). The odds of detecting *S. aureus* from cheese was significantly lower as compared to raw milk and yogurt (OR = 0.16, 95% CI: 0.04-0.70) (Table 1).

Occurrence of *S. aureus* in milk and other dairy products ranged from 0% in traditionally processed cheese and yogurt, to as high as 28.6% in milk samples. There was no statistically significant difference in isolation of *S. aureus* from milk and traditionally processed dairy products collected from different subcities (*p* > 0.05) (Table 2).

### 3.2. Antimicrobial Susceptibility Profiles of *Staphylococcus aureus* Isolates

This study demonstrated the existence of high levels of resistance to commonly used antimicrobials in *S. aureus* isolates from milk and other dairy products. Of the 52 *S. aureus* isolates tested for susceptibility to 12 antimicrobials, the highest rate of resistance was observed to ampicillin (*n* = 49, 94.2%) followed by amoxicillin-clavulanic acid (*n* = 42, 80.8%) and the lowest rate was noted for ceftriaxone (*n* = 1, 1.9%). Twenty-four (46.2%) of the *S. aureus* isolates were resistant to tetracycline, and 20 (38.5%) of isolates were resistant to sulfamethoxazole+trimethoprim. Resistance to cefoxitin, which was defined as methicillin-resistant *Staphylococcus aureus* (MRSA), was detected in 20 (38.5%) of the isolates (Table 3). However, only one (5%), of the 20 isolates, was found positive for the *mec*A gene, and none of the isolates were found to carry the *mec*C gene. The *mec*A-positive isolate was obtained from a raw milk sample collected from Kirkos Sub-city.

All of the 52 isolates were resistant to at least one of the antimicrobials tested, and resistance to two or more antimicrobials was identified in 50 (96.2%) of the isolates; resistance to three or more antimicrobials was detected in 39 (75%) of the isolates. Resistance to 5 or more number of antimicrobials was recorded in 13 (25%) of the isolates. The two frequently detected resistance patterns were resistant to ampicillin-amoxicillin+clavulanic acid, and ampicillin-amoxicillin+clavulanic acid-tetracycline in seven isolates each (Table 4).

## 4. Discussion

The current study identified that 24.6% of the raw milk and 11.3% of traditionally processed dairy products sampled from selected subcities in Addis Ababa were positive for *S. aureus.* The prevalence of *S. aureus* reported here is in agreement with a study conducted in Hawassa area, South Ethiopia, which reported that 25% of raw cow milk samples were positive for *S. aureus* [19]. Our study is also consistent with prevalence reported by other studies from central Ethiopia (21%) [20] and in Sebeta town (23.4%) [11]. Our study demonstrated a higher prevalence than the 16.6% prevalence reported for milk samples from dairy cattle in Mukaturi and Sululta towns of Oromia region, Ethiopia [21]. Another study conducted in Jimma, Ethiopia, demonstrated a higher prevalence of *S. aureus* (52%) in bovine milk [22]. A study in selected regions of Jimma showed 14.3% prevalence of *S. aureus* in cottage cheese and yogurt which is in agreement with the 11.3% findings of the current study [15]. A potenital reason why the prevalence of S. *aureus* in the current study was low compared to the previous study, could be due to the fact that dairy farmers, and milk and other dairy product handlers in the Addis Ababa, are in a better position regarding awareness of hygienic practices.

The contamination of raw milk with *S. aureus* was higher than yogurt and cottage cheese in the current study. Potential causes for this include, contamination of milk due to infection of the mammary glands in cows with subclinical mastitis, contamination from the environment, or due to poor hygienic practices during or after milking. Poor hygienic practices that have been associated with contaminated milk include, not washing hands when handling milk storage equipment [23]. The processing of dairy products, whether it is conducted traditionally or using modern techniques, might have potentially reduced the staphylococcal contamination through heat and fermentation processes [2]. Traditional preparation of yoghurt involves keeping the milk at room temperature until it ferments without pasteurization and addition of microorganisms for initiation of fermentation, whereas traditional cheese is made by heating milk from which butter is removed after churning [24].

Considerable resistance to antimicrobials was detected among *S. aureus* isolates in milk and other dairy products in this study particularly, high rate of resistance to ampicillin (94.2%) and amoxicillin+clavulanic acid (80.8%) was recorded. Similar high rate of resistance to ampicillin and amoxicillin+clavulanic acid was reported from previous studies in Ethiopia [3, 19]. This is probably due to frequent use of different *β*-lactams in the study areas contributing to selection for resistant strains. Similar high levels of resistance to tetracycline and sulfamethoxazole+trimethoprim in the current and previous studies could be due to overuse of these antimicrobials for treatment and prevention of various animal infections in the study area [11]. Oxytetracycline and antimicrobials belonging to the sulfonamide family are commonly prescribed antimicrobials in the animal health sector in Ethiopia [25, 26]. A potential reason why high levels of resistance to several antimicrobials was recorded in this study could be due to the fact that dairy farmers in urban and periurban areas of Addis Ababa have relatively high access to antimicrobials exposing their animals to antimicrobials frequently. This might have increased selection for resistant isolates. Previous study also reported similar high levels of MDR non-typhoidal *Salmonella* isolates from dairy farms in Addis Ababa as compared to isolates obtained from dairy cattle out of Addis Ababa [25]. In addition, close contact with the human population may also expose animals and milk samples to resistant organisms originating from human.

In our current study, over 38% of the *S. aureus* isolates were methicillin resistant phenotypically based on susceptibility to cefoxitin. This is lower than the previous study where 100% of *S. aureus* isolates from milk value chain around Sebeta were reported to be resistant to cefoxitin [11]. However, another study from North-West Ethiopia reported that none of the 79 *S. aureus* isolates from cows with intramammary infection were resistant to cefoxitin [3]. A potential explanation for such a huge difference could be due to the type of antimicrobial susceptibility tests used. Mekonnen et al. used broth microdilution assay whereas the current study, and previous study by Ayele et al. used disc diffusion assay. Cefoxitin disc diffusion test was reported to be 100% sensitive and 91.6% specific in detecting MRSA [27]. Interestingly, only one of the 20 phenotypically MRSA isolates (5%) was found positive for the *mec*A gene in the current study, and none were positive for the *mec*C gene. In a study conducted on *S. aureus* isolated from bovine milk samples in central Ethiopia, despite 53.2% prevalence of MRSA based on cefoxitin resistance, none of the 109 isolates tested were positive for the *mec*A gene [20]. Similarly, 139 *S. aureus* isolates with phenotypic resistance to oxacillin (methicillin) were all negative for the *mec*A gene using classical PCR detection in a study conducted in Nigeria [28]. A previous study from Sudan also reported the absence of the *mec*A gene in 9.8% of MRSA strains isolated from different clinical samples [29]. Detection of *mec*A or *mec*C genes has long been considered as a major confirmatory method for MRSA [18]. However, the absence of *mec*A or *mec*C genes in this study, and other studies, suggests the possibility of other mechanisms behind MRSA phenotype. Previous studies also reported similar findings, suggesting alternative genetic mechanisms [29]. A few recent studies also showed chromosomal and plasmid-mediated homologues of the *mec*A gene conferring resistance to methicillin (*mec*B and *mec*D) [30–32].

*Staphylococcus aureus* is one of the important foodborne pathogens that can cause a wide variety of diseases in humans, and its detection in milk and other dairy products poses serious public health risks [33]. There are significant public health impacts of *S. aureus*, with respect to healthcare costs, and length of hospital stay increases when the isolates are resistant to antimicrobials, particularly MRSA compared to susceptible strains [34]. Resistance to as many as seven antimicrobials was detected in one of the isolates in the current study. The observation of such considerable levels of resistance in the isolates, warrants the need for strong regulation and the prudent use of antimicrobials.

## 5. Conclusion

This study showed significant levels of contamination of milk and dairy products with *S. aureus*, and most of the isolates were multidrug resistant. Particularly, the occurrence of MRSA in raw milk and dairy products signifies a serious public health threat due to the widespread practice of consuming raw dairy products in the study area. The lack of concordance between phenotypic and genotypic detection of MRSA in the current study implies the need for further research to identify the genetic basis for such differences.

## Figures and Tables

**Table 1:** Prevalence of *Staphylococcus aureus* in raw milk, yogurt, and cottage cheese collected in Addis Ababa, Ethiopia.

Sample type	No. tested	No. (%) positive	*χ* ^2^	*p* value	OR (95% CI)
Raw milk	175	43 (24.6)	7.93	0.019	Reference
Yogurt	40	7 (17.5)			0.65 (0.27-1.58)
Cheese	40	2 (5)			0.16 (0.04-0.70)
Total	255	52 (20.4)			

**Table 2:** Occurrence of *Staphylococcus aureus* based on type of samples among subcities of Addis Ababa.

Sample type	Subcity	No. tested	No. (%) positive	*χ* ^2^	*p* value
Milk	Kirkos	79	22 (27.8)	2.61	0.679
	Arada	13	2 (15.4)		
	Yeka	30	7 (23.3)		
	Nifas Silk Lafto	25	4 (16)		
	Akaki Kality	28	8 (28.6)		

Total		175	43 (24.57)		

Yogurt	Kirkos	8	2 (25)	2.77	0.597
	Arada	8	2 (25)		
	Yeka	8	1 (12.5)		
	Nifas Silk Lafto	8	2 (25)		
	Akaki Kality	8	0		

Total		40	7 (17.5)		

Cheese	Kirkos	8	0	8.42	0.077
	Arada	8	2 (25)		
	Yeka	8	0		
	Nifas Silk Lafto	8	0		
	Akaki Kality	8	0		

Total		40	2 (5)		

**Table 3:** Antimicrobial susceptibility profile of *Staphylococcus aureus* isolates from raw milk and other dairy products in Addis Ababa, Ethiopia (*n* = 52).

Name of antimicrobial	Susceptibility patterns		
	Susceptible (No., %)	Intermediate (No., %)	Resistant (No., %)
Cefoxitin	32 (61.5)	—	20 (38.5)
Tetracycline	26 (50)	2 (3.8)	24 (46.2)
Erythromycin	36 (69.2)	3 (5.8)	13 (25)
Amoxicillin	44 (84.6)	6 (11.5)	2 (3.9)
Ceftriaxone	46 (88.5)	5 (9.6)	1 (1.9)
Amoxicillin+clavulanic acid	10 (19.2)	—	42 (80.8)
Ampicillin	3 (5.8)	—	49 (94.2)
Ciprofloxacin	46 (88.5)	4 (7.7)	2 (3.9)
Cephalothin	39 (75)	5 (9.6)	8 (15.4)
Chloramphenicol	46 (88.5)	4 (7.7)	2 (3.9)
Gentamicin	38 (73.1)	9 (17.3)	5 (9.6)
Sulfamethoxazole+trimethoprim	32 (61.5)	—	20 (38.5)

**Table 4:** Antimicrobial resistance pattern of *Staphylococcus aureus* isolates in milk and other dairy products in Addis Ababa, Ethiopia.

No. of antimicrobials to which isolates were resistant	Resistance pattern	No.	%
One	AM	2	3.9

Two	AM, AMC	7	13.5
	AM, TE	1	1.9
	TE, SXT	1	1.9
	AM, SXT	1	1.9
	AM, E	1	1.9

Three	AM, AMC, TE	7	13.5
	AMC, TE, SXT	1	1.9
	AM, AMC, SXT	1	1.9
	AM, AN, SXT	1	1.9

Four	AM, AMC, TE, SXT	3	5.8
	AM, AMC, FOX, TE	4	7.7
	AM, AMC, FOX, SXT	4	7.7
	AM, AMC, GM, SXT	1	1.9
	AM, AMC, CIP, SXT	1	1.9
	AM, AMC, FOX, GM	1	1.9
	AM, AMC, FOX, E,	1	1.9
	AM, AMC, CF, E	1	1.9

Five	AM, AMC, AN, CIP, SXT	1	1.9
	AM, AMC, CF, E, TE,	1	1.9
	AM,AMC, FOX, E, TE,	1	1.9
	AM, AMC, C, TE, SXT	1	1.9
	AM, AMC, E, TE, SXT	1	1.9
	AM, AMC, CF, CXT, E,	1	1.9

Six	AM, AMC, CF, FOX, E, SXT	2	3.9
	AM, AMC, FOX, E, CF, GM	2	3.9
	AM, AMC, FOX, TE, C, SXT	1	1.9
	AM, AMC, CXT, E, TE, SXT	1	1.9

Seven	AM, AMC, CRO, CF, FOX, TE, SXT	1	1.9

Total		52	100

FOX: cefoxitin; CRO: ceftriaxone; AMC: amoxicillin+clavulanic acid; GN: gentamicin; C: chloramphenicol; CIP: ciprofloxacin; AMP: ampicillin; CF: cephalothin; TE: tetracycline; AN: amoxicillin; ER: erythromycin; SXT: sulfamethoxazole+trimethoprim.

## Data Availability

The data used to support the findings of this study are included within the article.

## References

[1] Pereira P. C. (2014). Milk nutritional composition and its role in human health. *Nutrition*.

[2] Dhanashekar R., Akkinepalli S., Nellutla A. (2012). Milk-borne infections. An analysis of their potential effect on the milk industry. *Germs*.

[3] Mekonnen S. A., Lam T., Hoekstra J. (2018). Characterization of Staphylococcus aureus isolated from milk samples of dairy cows in small holder farms of North-Western Ethiopia. *BMC Veterinary Research*.

[4] Cremonesi P., Pozzi F., Raschetti M. (2015). Genomic characteristics of Staphylococcus aureus strains associated with high within-herd prevalence of intramammary infections in dairy cows. *Journal of Dairy Science*.

[5] McMillan K., Moore S. C., McAuley C. M., Fegan N., Fox E. M. (2016). Characterization of Staphylococcus aureus isolates from raw milk sources in Victoria, Australia. *BMC Microbiology*.

[6] Grispoldi L., Massetti L., Sechi P. (2019). Short communication: characterization of enterotoxin-producing Staphylococcus aureus isolated from mastitic cows. *Journal of Dairy Science*.

[7] Goncalves J. L., Kamphuis C., Vernooij H. (2020). Pathogen effects on milk yield and composition in chronic subclinical mastitis in dairy cows. *Veterinary Journal*.

[8] Grispoldi L., Karama M., Armani A., Hadjicharalambous C., Cenci-Goga B. T. (2021). Staphylococcus aureus enterotoxin in food of animal origin and staphylococcal food poisoning risk assessment from farm to table. *Italian Journal of Animal Science*.

[9] Hammad A. M., Watanabe W., Fujii T., Shimamoto T. (2012). Occurrence and characteristics of methicillin-resistant and -susceptible Staphylococcus aureus and methicillin-resistant coagulase-negative staphylococci from Japanese retail ready-to-eat raw fish. *International Journal of Food Microbiology*.

[10] Lee A. S., de Lencastre H., Garau J. (2018). Methicillin-resistant Staphylococcus aureus. *Nature Reviews. Disease Primers*.

[11] Ayele Y., Gutema F. D., Edao B. M. (2017). Assessment of Staphylococcus aureus along milk value chain and its public health importance in Sebeta, central Oromia, Ethiopia. *BMC Microbiology*.

[12] CDO (2021). *Addis Abeba climate (Ethiopia)*.

[13] Thrusfield M. (2007). *Veterinary Epidemiology, Third Edition*.

[14] Megersa L. (2018). *Identification and antimicrobial susceptibility of Staphylococcus species isolated from raw milk, udder swabs, milking utensils and milkers hands in small holder and dairy farms in Ambo and Guder towns*.

[15] Argaw S., Addis M., Degefu H. (2018). Identification and antimicrobial resistance pattern of staphylococci isolated from cottage cheese (Ayib) and yoghurt (Ergo) in selected districts of Jimma Zone, Ethiopia. *Health Science Journal*.

[16] CLSI (2018). *M100: performance standards for antimicrobial susceptibility testing: twenty-third informational supplement*.

[17] Jain A., Agarwal A., Verma R. K. (2008). Cefoxitin disc diffusion test for detection of meticillin-resistant staphylococci. *Journal of Medical Microbiology*.

[18] Stegger M., Andersen P. S., Kearns A. (2012). Rapid detection, differentiation and typing of methicillin-resistant Staphylococcus aureus harbouring either mecA or the new mecA homologue mecA(LGA251). *Clinical Microbiology and Infection*.

[19] Daka D., G/silassie S., Yihdego D. (2012). Antibiotic-resistance Staphylococcus aureus isolated from cow's milk in the Hawassa area, South Ethiopia. *Annals of Clinical Microbiology and Antimicrobials*.

[20] Tigabu E., Kassa T., Asrat D. (2015). Phenotypic and genotypic characterization of Staphylococcus aureus isolates recovered from bovine milk in central highlands of Ethiopia. *African Journal of Microbiology Research*.

[21] Regasa S., Mengistu S., Abraha A. (2019). Milk safety assessment, isolation, and antimicrobial susceptibility profile of Staphylococcus aureus in selected dairy farms of Mukaturi and Sululta town, Oromia region, Ethiopia. *Veterinary medicine international*.

[22] Sori T., Hussien J., Bitew M. (2011). Prevalence and susceptibility assay of Staphylococcus aureus isolated from bovine mastitis in dairy farms of Jimma town, South West Ethiopia. *Journal of Animal and Veterinary Advances*.

[23] Quigley L., O'Sullivan O., Stanton C. (2013). The complex microbiota of raw milk. *FEMS Microbiology Reviews*.

[24] Berhe T., Vogensen F. K., Ipsen R., Seifu E., Kurtu M. Y., Hanse E. B. (2017). Traditional fermented dairy products of Ethiopia: a review. *East African Journal of Sciences*.

[25] Eguale T., Engidawork E., Gebreyes W. A. (2016). Fecal prevalence, serotype distribution and antimicrobial resistance of Salmonellae in dairy cattle in central Ethiopia. *BMC Microbiology*.

[26] Beyene T., Endalamaw D., Tolossa Y., Feyisa A. (2015). Evaluation of rational use of veterinary drugs especially antimicrobials and anthelmintics in Bishoftu, Central Ethiopia. *BMC Research Notes*.

[27] Pourmand M. R., Hassanzadeh S., Mashhadi R., Askari E. (2014). Comparison of four diagnostic methods for detection of methicillin resistant Staphylococcus aureus. *Iranian Journal of Microbiology*.

[28] Olayinka B. O., Olayinka A. T., Obajuluwa A. F., Onaolapo J. A., Olurinola P. F. (2009). Absence of mecA gene in methicillin-resistant Staphylococcus oureus isolates. *African Journal of Infectious Diseases*.

[29] Elhassan M. M., Ozbak H. A., Hemeg H. A., Elmekki M. A., Ahmed L. M. (2015). Absence of the mecA gene in methicillin resistant Staphylococcus aureus isolated from different clinical specimens in Shendi City, Sudan. *BioMed Research International*.

[30] Lakhundi S., Zhang K. (2018). Methicillin-resistant Staphylococcus aureus: molecular characterization, evolution, and epidemiology. *Clinical Microbiology Reviews*.

[31] Becker K., van Alen S., Idelevich E. A. (2018). Plasmid-encoded transferable mecB-mediated methicillin resistance in Staphylococcus aureus. *Emerging Infectious Diseases*.

[32] Andreis S. N., Perreten V., Schwendener S. (2017). Novel *β*-LactamaseblaARLin Staphylococcus arlettae. *mSphere*.

[33] Bintsis T. (2017). Foodborne pathogens. *AIMS Microbiology*.

[34] Zhen X., Lundborg C. S., Zhang M. (2020). Clinical and economic impact of methicillin-resistant *Staphylococcus aureus*: a multicentre study in China. *Scientific Reports*.

